# Medical Fees in America

**Published:** 1865-02

**Authors:** 


Medical Fees in America.—The "doctors" of Hanover county, Virginia, announce the following as their tariff of fees:—Thirty dollars per visit for all distances of five miles and under; all distances over five miles, an additional fee of two dollars per mile; night visits double that of day visits; consultation fee, forty dollars; obstetric cases, one hundred dollars.—*Lancet.*

